# Reliability of a multi-segment foot model in a neutral cushioning shoe during treadmill walking

**DOI:** 10.1186/s13047-018-0301-2

**Published:** 2018-11-14

**Authors:** Megan E. R. Balsdon, Colin E. Dombroski

**Affiliations:** 1 SoleScience Inc., Fowler Kennedy Sports Medicine Clinic, 3M Building, Western University, London, ON N6A 3K7 Canada; 2 Faculty of Health Science, Department of Physical Therapy, Western University, London, ON N6A 3K7 Canada

**Keywords:** Multi-segment foot model, Oxford foot model, Foot kinematics, Motion capture, Neutral cushioning walking shoe, Gait

## Abstract

**Background:**

Detailed kinematics of the foot has been frequently reported on in the literature, specifically using various multi-segment foot models. It is important to identify the reliability of a multi-segment foot model in a population of mixed genders and activity levels, while walking in commonly used footwear. The main objective of this study was to investigate the between-day reliability and within-session variability of the Oxford Foot Model (OFM) while walking in a neutral cushioning shoe.

**Methods:**

A 7-camera Vicon motion capture system was used along with 29 passive reflective markers, placed on the participant to examine the multi-segment foot kinematics of the left foot using the OFM. Windows were cut in New Balance 840 shoes following reports from a previous investigation to maintain shoe integrity during testing. Two walking sessions on separate days were collected for 12 healthy participants, with an average total of 22 gait cycles per day.

**Results:**

ICCs ranged from 0.020 to 0.964 for between-day reliability, and within-session ICC values ranged from 0.268 to 0.985. Between-day ICC values of the relative measures (excursion and range of motion (ROM)) were higher than the absolute angle measures (angle at foot strike and peak angle). Largest differences were measured in the transverse plane, and the smallest differences in the sagittal plane. Bland-Altman plots revealed best agreement in the frontal and sagittal planes. SEM values ranged from 0.04 to 3.5 for the between-day reliability.

**Conclusions:**

Between-day reliability and within-session variability were comparable to previous studies for adults walking barefoot and shod. This research demonstrates that the OFM can produce reliable data when applied to the assessment of a shod foot.

## Background

Detailed kinematics of the foot has been frequently reported in the literature; specifically, multi-segment foot models using optical motion capture have been developed to measure kinematics of up to four foot segments [1–3]. Multi-segment foot models are used as clinical tools for a more precise analysis of foot kinematics in both healthy and pathological populations. The Oxford Foot Model (OFM) has been used to measure kinematics of three foot segments: the hindfoot, forefoot and hallux [4]. This model has specifically been used to describe both normal and pathological gait of children [5–7] and adults [8, 9].

Foot kinematics have been previously measured using shoe-based markers, with one study reporting that tracking markers attached to the shoe overestimates rearfoot motion, compared to markers placed directly on the calcaneus [10]. When multi-segment foot kinematics are measured in a walking or running shoe, the chosen footwear must be modified in order to place the markers directly on the skin. However, large incisions in the shoe may affect its structure, integrity and support that it will provide for the foot. Shultz & Jenkyn [11] investigated hole sizes in a running shoe that would maintain the integrity of the shoe, the maximum hole size was found to be an oval of 2.7 cm by 2.3 cm [11]. A second study investigated and confirmed the appropriate hole size in a walking shoe by demonstrating that holes with a diameter of 2.5 cm were large enough to allow free motion of marker wands mounted on the skin surface during walking [12].

One study has performed an analysis with the OFM in a running shoe with holes of 2.5 cm in diameter in order to compare foot orthotic conditions [13]. Another group used a similar model to the OFM while walking and running in sandals of two degrees of hardness at the midfoot (soft and hard); however, the sandals did not require any modifications to complete the study [14]. Despite ease of use in applying the markers, the sandals are not representative of the shoes people actually wear in daily living or sporting applications.

Evidence of adequate reliability for using the OFM while walking barefoot has been reported in the literature in healthy adults [4, 15] and in children [16, 17]. More recently, the reliability and minimal detectable difference in a modified OFM was investigated during shod walking and running in active, healthy adult men [18]. There is a gap in the literature evaluating the reliability of the OFM in a group with unspecified activity levels and mixed genders. Additionally, the literature reporting on the reliability of multi-segment foot models in footwear is limited, and more specifically, the reliability of the OFM in a neutral cushioning walking shoe is unknown. The main objective of this study was to investigate the between-day reliability and within-session variability using the OFM while walking in a neutral cushioning shoe. A test-retest reliability study using a healthy mixed population will help to understand the variability in this specific multi-segment kinematic foot model, while also assessing the motion of the foot within the shoe.

## Methods

### Participants

Twelve healthy volunteers (4 male, 8 female) (mean 24 ± 6.9 yrs., 170.5 ± 12.9 cm, 72.3 ± 13.9 kg) with no current injuries took part in the study. All procedures received prior approval from the appropriate ethics board and all volunteers provided written informed consent. Participants were recruited from a university population via posted flyers. Inclusion criteria for participation included only that the participants were between 18 and 65 years of age.

### Experimental protocol

**Fig. 1:**
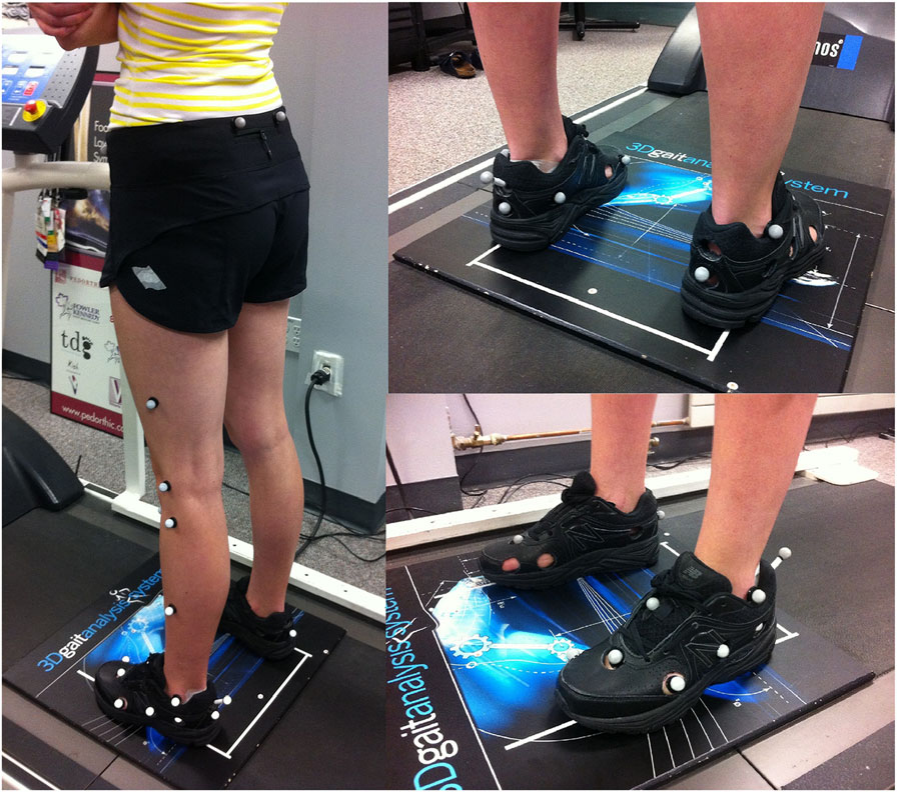
Participant standing during static stance collection for lower body gait with the Oxford Foot Model on the left foot only. Worn by the participant are the New Balance neutral cushioning shoes (model 840 – black leather)

Participants were assessed on two separate days, at least one week apart. The assessor, a research assistant with three years of experience with this specific Vicon motion capture system and an additional two years of biomechanics experience, collected all participants’ gait data for both sessions. Participants walked on a motorized treadmill (Impulse-Pro RT500, Impulse Health Tech Co. Ltd., Midlothian, Scotland) for a total acclimatization period of three minutes and then trials were recorded at the volunteers’ self-selected speed (average 1.09 ± 0.13 m/s). Each participant’s speed was repeated during the second testing session. Three walking trials were collected for both testing sessions with approximately 6–10 gait cycles per trial. Footwear was controlled by using the New Balance neutral cushioning shoe (model 840) with circular holes cut out with a diameter of 2.5–2.7 cm based on findings from Shultz & Jenkyn [11] and Bishop et al. [12]. The markers were applied to the skin in the locations originally defined by Carson et al. (2001) which include the shank, hindfoot, forefoot and hallux segments (Fig. 1). A more detailed description of marker placement is outlined and illustrated in Stebbins et al. [16]. All markers were 14 mm in diameter, attached directly on the skin with double-sided adhesive and there was a single 14 mm wand marker on the posterior calcaneus, similar to Stebbins et al. [16].

### Methodology

A 7-camera (Bonita B3) motion capture system (Vicon Motion Systems Ltd., Oxford, UK), along with 29 passive reflective markers, was used to collect gait kinematics of the lower body and left foot of each participant. Markers were carefully placed on the landmarks defined the Oxford Foot Model (OFM) [4]. Marker data was collected at 200 Hz. A static standing trial was first collected for calibration purposes and specific model creation of each individual. The static position was standard for each participant, with lines marked on a board that was placed on the treadmill and each participant lined up their feet on the lines for consistency. Measurements chosen for analysis were hindfoot motion with respect to the tibia, forefoot motion with respect to the hindfoot and hallux motion with respect to the forefoot in the sagittal plane only. The parameters extracted and presented for each of these measurements include the angle at foot strike, peak angle during the first 60% of stance phase, the excursion from foot strike to peak, and finally, range of motion during the entire stance phase.

### Data processing

Following successful data collection of approximately 30 gait cycles per subject, processing of trial data was first performed using Vicon Nexus (Vicon, Oxford Metrics Ltd., Oxford, UK). Reconstruction and labelling of marker trajectories was first performed, followed by gaps in the trajectories that were automatically filled using the Woltring operation. Any remaining small gaps (marker occlusion of up to 5 frames) were manually filled with a quintic spline or the pattern fill option in Nexus (version 1.8.5). Since no kinetic measures were collected, gait cycle events of initial contact and foot off were manually inputted by the user based on visual assessment of the foot relative to the ground. The dynamic gait OFM operation was run for each trial, which were then exported into .csv files. Each gait cycle was interpolated to 100% of gait, and then averaged for each trial and each day. Gait cycles that included points outside two standard deviations (SDs) from the median during stance phase were considered outliers and were removed from the analysis. We chose this method to remove cycles that would likely be considered a ‘miss-step’. This resulted in an average of 22 gait cycles for the between-day analysis. Once the gait cycles were processed and averaged, the measured parameters from stance phase of the gait cycle could be extracted and analyzed. The average of each parameter for Day 1 was compared to the average for Day 2 for the between-day reliability analysis, whereas the within-session analysis compared three averaged trials.

### Statistical analysis

Intraclass correlation coefficients were used to determine the between-day (ICC(3,k)) and within-session (ICC(3,1)) reliability using a two-way mixed analysis for absolute agreement (SPSS Inc., IBM Corporation), where ‘k’ is the number of trials used to obtain the mean. To determine validity of the ICC for the between-subjects effect, the F-test was used with a *p* < 0.05 cut-off to include a heterogeneous group of data. The ICC scale used was the same as the one used in Wright et al. (2011), where less than 0.4 was considered poor, between 0.4 and 0.75 was fair to good and greater than 0.75 was considered excellent reliability. Standard Error of Measurement (SEM) was calculated using one of its basic forms [SEM = SD√(1-ICC)], and the Root Mean Square Error was calculated for the between-day reliability $RMSE=\sqrt{\frac{\sum {\left({M}_{d1}-{M}_{d2}\right)}^2}{n}}$. Bland-Altman plots were also used to assess the repeatability of the OFM, plotting the average of the between-day testing sessions (x-axis) against the difference between sessions (y-axis) for all three joints studied [19].

## Results

**Fig. 2:**
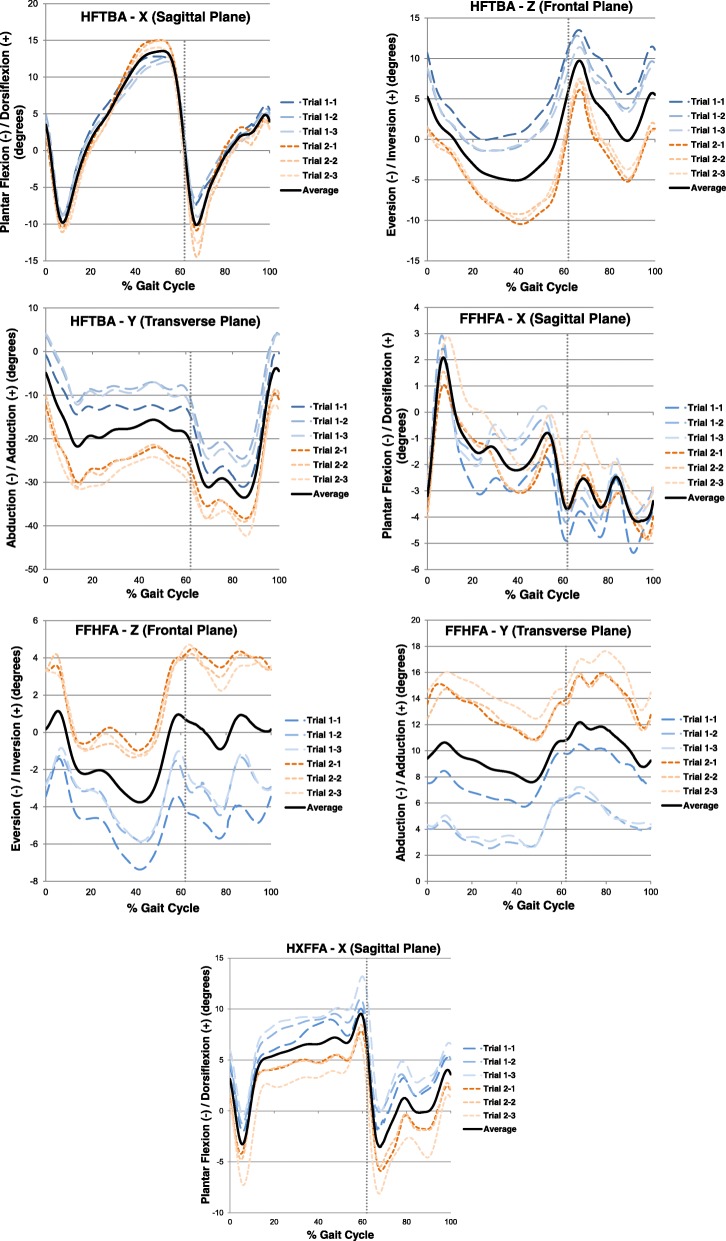
Example trial data for a female, 20 yrs., 163 cm, and 60 kg, women’s size 7 shoe, with a normal foot posture. Data shows hindfoot motion with respect to the tibia (HFTBA) and forefoot with respect to the hindfoot (FFHFA), both in the Sagittal (X), Frontal (Z), and Transverse (Y) Planes. The hallux with respect to the hindfoot (HXFFA) is shown in the Sagittal Plane (X) only. Day 1 is represented in the blue long dashes and Day 2 is represented in the orange square dotted lines. Dotted vertical line represents the end of stance phase and beginning of swing phase at 62% of the gait cycle

Four (4) foot kinematic parameters for the hindfoot and forefoot segments were compared both within-session and between-day for three planes of motion where the hallux segment was evaluated only in the sagittal plane. Upon visual inspection, the patterns of movement were found to be consistent, with some offsets observed, mostly between days (Fig. 2).

ICCs ranged from 0.020 to 0.964 for between day reliability, excluding negative ICCs (Table 1). The standard error measurement (SEM) range was between 0.04 to 3.5 and the root mean squared error (deviation) ranged from 0.73 to 16.2 degrees, with the two largest values in the transverse plane. Within-session ICC values ranged from 0.268 to 0.985, with only one ICC with *p* > 0.05 (Table 2). The between-day ICC values for the excursion and range of motion (ROM) measures appeared to be slightly higher than the absolute measures such as angle at foot strike and peak values, whereas the ICC values for within-session reliability appeared similar between relative and absolute measures.

**Table 1:** Between-day reliability of the hindfoot, forefoot and hallux during walking

Segment	Plane	Angle	Mean (deg)		ICC	SEM	RMSE (deg)
			Day 1 (SD)	Day 2 (SD)			
Hindfoot-tibia	Sagittal	Foot Strike	4.9 (4.0)	3.0 (3.3)	−0.556	2.8	6.5
		Peak DF	12.7 (5.7)	10.7 (3.0)	−0.104	2.4	6.8
		DF Excursion	19.7 (3.7)	19.8 (1.8)	0.250	0.2	3.5
		ROM	25.4 (4.4)	24.7 (2.4)	0.111	0.7	4.6
	Frontal	Foot Strike	−0.64 (8.0)	1.1 (4.9)	0.449	1.5	6.9
		Peak EVE	−10.4 (8.0)	−8.4 (4.6)	0.345	1.9	7.4
		EVE Excursion	11.3 (3.6)	10.1 (2.3)	0.495*	1.0	3.1
		ROM	16.0 (5.0)	14.2 (3.3)	0.020	2.0	5.9
	Transverse	Foot Strike	−9.8 (13.4)	−6.3 (12.0)	0.272	3.5	15.2
		Peak ABD	−25.6 (12.7)	−22.8 (10.4)	−0.032	3.3	16.2
		ABD Excursion	15.9 (5.5)	16.8 (4.4)	0.345	0.9	5.6
		ROM	19.1 (4.9)	21.6 (7.4)	0.168	2.6	8.1
Forefoot-hindfoot	Sagittal	Foot Strike	−7.8 (3.8)	−6.2 (4.0)	−0.042	1.9	5.7
		Peak DF	−1.8 (3.9)	−0.25 (4.7)	0.183	1.6	5.5
		DF Excursion	7.0 (3.3)	6.7 (2.4)	0.665*	0.2	2.3
		ROM	8.9 (3.2)	8.2 (3.0)	0.528*	0.5	3.0
	Frontal	Foot Strike	4.2 (6.5)	3.3 (3.9)	0.477*	0.7	5.4
		Peak EVE	0.72 (6.3)	−0.16 (4.1)	0.558*	0.7	4.9
		EVE Excursion	5.2 (1.5)	5.4 (1.8)	0.592*	0.2	1.5
		ROM	5.8 (1.2)	6.3 (1.4)	0.054	0.6	1.8
	Transverse	Foot Strike	8.6 (6.4)	10.9 (4.0)	0.108	2.5	7.2
		Peak ABD	6.9 (6.5)	9.2 (4.0)	0.149	2.4	7.2
		ABD Excursion	2.4 (0.7)	2.5 (1.0)	0.305	0.0	1.0
		ROM	3.6 (0.7)	3.7 (1.2)	0.710*	0.1	0.7
Hallux-forefoot	Sagittal	Foot Strike	4.3 (6.5)	4.6 (9.5)	0.725*	0.1	6.0
		Peak DF	8.7 (5.3)	8.9 (7.8)	0.674*	0.1	5.3
		DF Excursion	9.7 (6.1)	9.6 (5.4)	0.949*	0.0	1.8
		ROM	14.4 (7.2)	15.1 (7.2)	0.964*	0.1	1.9

DF - Dorsiflexion, EVE - Eversion, ABD - Abduction, ROM - Range of Motion, * *p*-value < 0.05

**Table 2:** Within-session reliability of the hindfoot, forefoot and hallux during walking

Segment	Plane	Angle	Mean (deg)			ICC	SEM
			Trial 1 (SD)	Trial 2 (SD)	Trial 3 (SD)		
Hindfoot-tibia	Sagittal	Foot Strike	5.2 (3.7)	4.9 (3.7)	5.2 (4.3)	0.802*	0.1
		Peak DF	13.1 (4.9)	12.5 (5.5)	12.9 (6.0)	0.897*	0.1
		DF Excursion	19.4 (3.6)	20.3 (4.1)	19.6 (4.1)	0.876*	0.3
		ROM	25.6 (4.5)	26.1 (4.1)	25.3 (4.3)	0.906*	0.2
	Frontal	Foot Strike	−0.82 (8.5)	−1.0 (8.2)	−0.26 (7.9)	0.976*	0.1
		Peak EVE	−11.2 (8.6)	−10.9 (8.2)	−9.7 (7.7)	0.980*	0.2
		EVE Excursion	11.8 (3.6)	11.8 (3.9)	11.0 (3.8)	0.929*	0.2
		ROM	16.5 (5.1)	16.6 (5.0)	15.3 (4.6)	0.945*	0.3
	Transverse	Foot Strike	−11.0 (13.1)	− 9.3 (13.3)	− 9.6 (13.4)	0.984*	0.2
		Peak ABD	−26.7 (12.8)	−25.7 (13.1)	−24.9 (12.3)	0.985*	0.2
		ABD Excursion	15.8 (5.9)	16.5 (5.7)	15.3 (5.5)	0.930*	0.3
		ROM	19.2 (5.0)	19.6 (5.4)	18.6 (5.0)	0.903*	0.3
Forefoot-hindfoot	Sagittal	Foot Strike	−8.0 (4.1)	−6.7 (3.2)	−8.4 (4.4)	0.654*	0.8
		Peak DF	−2.3 (5.0)	−0.48 (3.5)	−2.5 (4.5)	0.620*	1.1
		DF Excursion	6.8 (3.3)	7.5 (3.5)	7.0 (3.4)	0.943*	0.1
		ROM	8.8 (3.1)	9.2 (3.0)	8.9 (3.6)	0.876*	0.1
	Frontal	Foot Strike	4.6 (6.9)	4.7 (7.3)	4.0 (6.5)	0.973*	0.1
		Peak EVE	1.0 (6.9)	1.1 (7.2)	0.47 (6.1)	0.967*	0.1
		EVE Excursion	5.3 (1.5)	5.3 (1.5)	5.3 (1.7)	0.869*	0.0
		ROM	5.9 (1.4)	6.1 (1.7)	6.0 (1.3)	0.800*	0.1
	Transverse	Foot Strike	9.5 (5.3)	8.8 (6.3)	8.5 (6.5)	0.979*	0.1
		Peak ABD	7.5 (6.8)	6.8 (6.7)	6.8 (6.2)	0.981*	0.1
		ABD Excursion	3.0 (1.6)	2.8 (1.1)	2.4 (0.9)	0.268	0.4
		ROM	4.3 (2.0)	4.1 (1.3)	4.0 (0.9)	0.395*	0.2
Hallux-forefoot	Sagittal	Foot Strike	5.1 (6.9)	4.9 (6.9)	4.8 (6.7)	0.989*	0.0
		Peak DF	9.4 (5.9)	9.3 (6.0)	9.8 (5.9)	0.983*	0.1
		DF Excursion	9.1 (6.0)	9.8 (6.9)	10.2 (6.7)	0.968*	0.2
		ROM	14.5 (8.1)	14.7 (7.9)	15.5 (7.8)	0.964*	0.2

DF - Dorsiflexion, EVE - Eversion, ABD - Abduction, ROM - Range of Motion, * *p*-value < 0.05

**Fig. 3:**
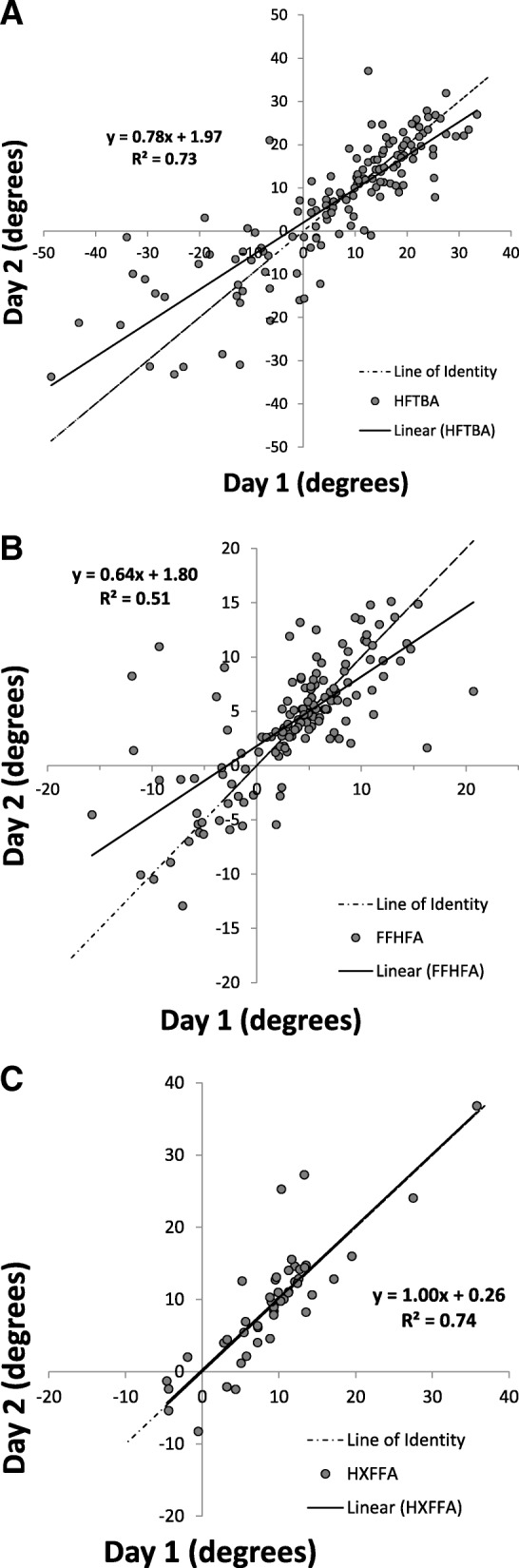
Scatter plots of Oxford Food Model results for the hindfoot (**a**), forefoot (**b**), and hallux (**c**) for Day 1 vs. Day 2 measurements, in degrees. The identity line (X = Y) is indicated in a dotted grey line

**Fig. 4:**
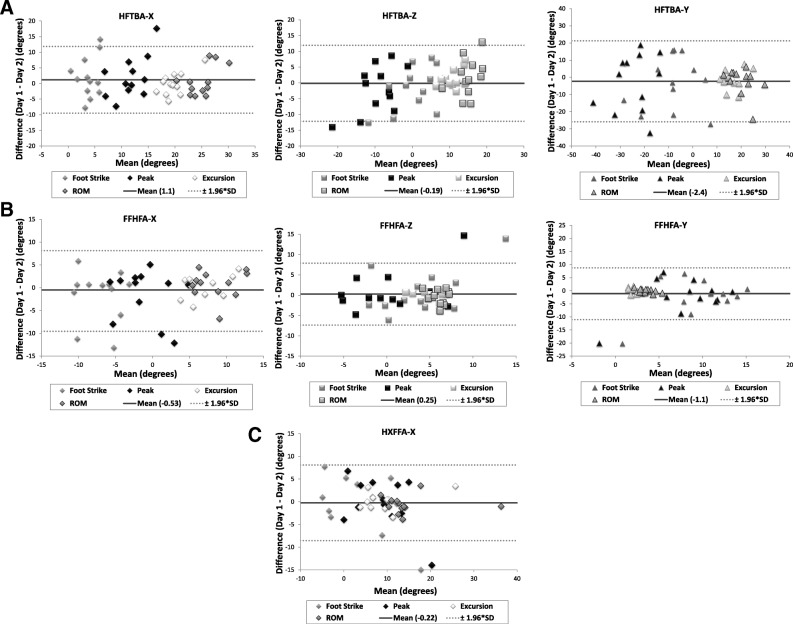
Bland-Altman plots of data for between-day testing comparison for the (**a**) hindfoot in all three planes (HFTBA-X, HFTBA-Z, HFTBA-Y), (**b**) forefoot in three planes (FFHFA-X, FFHFA-Z, FFHFA-Y), and (**c**) hallux in the sagittal plane (HXFFA-X). The mean difference in testing days is represented by the solid line, with the limits of agreement (±1.96*SD) represented by the dotted horizontal lines

The mean values for all of the measures for Day 1 vs. Day 2 were compared on scatter plots for all joints: **hindfoot** with respect to the tibia (HFTBA), **forefoot** with respect to the hindfoot (FFHFA) and the **hallux** with respect to the forefoot (HXFFA) (Fig. 3). The relationships show good correlation with R^2^ values of 0.73, 0.51 and 0.74, respectively. However, only the values for the hallux represented good agreement with the line of identity (X = Y), as the slope of the line is 1.0. Bland-Altman plots for the three joints, in each of the three planes, demonstrate the measure of repeatability for the between-day measurements (Fig. 4). The horizontal axis is the mean value of each of the measurements, whereas the vertical axis represents the difference of the measures for each day (i.e. Day 1-Day 2). The mean difference between the two measurements for each of the measures is shown as a solid horizontal line with the dotted horizontal lines representing ±1.96 standard deviations (SD) from the mean called the limits of agreement. For the hindfoot, the mean difference between days was 1.1° in the sagittal plane, − 0.19° in the frontal plane and − 2.4° in the transverse plane. In the forefoot, the mean differences were − 0.53°, 0.25°, and − 1.1° in the sagittal, frontal and transverse planes, respectively. The hallux showed a mean difference of − 0.22° in the sagittal plane.

## Discussion

The purpose of this study was to determine the reliability of the OFM during shod walking. Both between-day reliability and within-session variability was calculated for measures of the hindfoot with respect to the tibia, forefoot with respect to the hindfoot and the hallux with respect to the forefoot. The reliability of the OFM for walking in a neutral cushioning running shoe was determined using ICCs for absolute agreement, and the repeatability between days was also demonstrated using a Bland-Altman plot.

There was a fairly large range in reliability for many of the between-day measures in all three anatomical planes when looking at ICC values. Reliability between days was typically greatest in the sagittal plane, which was visually apparent in the case example (Fig. 2), and is consistent with previous studies measuring reliability of barefoot walking using the OFM [15–17]. Within-session variability was visually the most variable in the transverse plane. The transverse plane proved to be the least reliable between days with respect to the ICCs, where the literature has mixed results with both the frontal and transverse planes showing lower reliability [15–17]. Milner & Brindle (2016) performed intra-rater reliability for shod walking and running, and found that the relative measures such as excursion and range of motion showed better reliability compared to the absolute measures such as angle at foot strike and peak values. These findings are consistent with the present study findings. The range of SEM values (0.04–3.5) are also comparable to the range of values in previous literature (0.6–3.6) [18].

To strengthen the analysis of the repeatability of the measurement between days, the agreement between testing days was also measured using both regression and Bland-Altman plots. The regression plots showed the data was highly correlated (R^2^ values above 0.50), but found that only the hallux showed excellent agreement, with a slope close to 1 and a y-intercept of 0, indicating the measurements taken on the first occasion are similar to those taken on the second [20]. Since this regression analysis included data points from all of the variables (relative and absolute), this type of plot does not tell the whole story. The Bland-Altman plots show all variables divided by joint in all three planes. By joint, the mean differences were overall smaller in the forefoot and hallux, compared to the hindfoot. The hindfoot showed larger limits of agreement, but generally has much larger ranges of motion overall, in all three planes, compared to the forefoot and the hallux; thus, a larger standard deviation is expected. By plane, the transverse plane had the largest mean difference, and the frontal plane had the smallest, excluding the hallux as frontal plane motion was not measured. This finding differs from that of the ICCs, from both this study and others, that shows the sagittal plane as most reliable. The Bland-Altman plots provide a better description for each of the joints in all three planes, demonstrating that the majority of the data lie within the limits of agreement. All but one of the data points that lie outside of the limits of agreement were either the angle at foot strike or peak angle values, both absolute rather than relative values. This finding is consistent with the ICC values and previous literature [18]. Although the results from this type of analysis has not been done previously in the OFM literature, it provides a means for comparing the successive measurements between days.

It is understood among biomechanists that the reliability of marker placement on bony anatomy contributes to the variability in gait kinematics from optical motion capture. Wright et al. (2011) conducted both sessions, collecting the OFM on healthy adults one hour apart and the results of their absolute measures at initial contact and toe-off were only slightly better than longitudinal studies, which includes both marker re-placement and day-to-day variability. This indicates that the location of the markers on the skin after the first application will result in a more accurate comparison when the two conditions are tested on the same day, which is why the average ICC values appear slightly higher compared to our study, averaging a week between testing sessions.

The number of trials used for averaging each of the participants data was high compared to standard gait analysis research – an average of 22 cycles per trial for the between-day analysis. The majority of test-retest reliability studies use 3–10 cycles per session, with one particular study choosing only 3 strides after visually identifying the best of all traces of the session, which presents some bias [16]. A recent study found that approximately 23 strides on average should be captured to attain a reliable characteristic phase coordination index in healthy young adults [21]. The phase coordination index is a temporal measure that assesses bilateral coordination of gait. This finding suggests that a larger number of strides may be ideal for studies involving gait analysis, which shows strength in our study’s protocol. No study is without some limitations - this one is no different. The holes in the shoes provided were cut in advance, based on the anatomy of an individual with the correct foot size for each shoe; however, people with the same foot size may not have the same exact anatomy of their foot bones with respect to size, length and shape. Therefore, the main limitation is that the center of the holes cut in the shoe may not always align precisely with the bony anatomy of each person’s foot. From a cost perspective, it was not feasible to have a pair of shoes available for each participant for this study. Since the marker size was 14 mm and the shoe hole sizes were approximately 25 mm, it was difficult to move the marker too much within’ the pre-cut hole. Additionally, any motion of the foot inside the shoe leaves only 11 mm of total motion – 5-6 mm around the marker if it is completely centered – before the shoe interacts with the marker. Therefore, another limitation of this study is the likelihood of the marker and shoe interaction, especially with the increased depth of the heel counter in a walking shoe. Small 9 mm markers for the forefoot and midfoot, as well as single wand markers for the heel, such as those used in combined taping and foot orthotics study, will be the best solution for future studies in a running or walking shoe [22]. Finally, because there were no kinetic inputs from the treadmill, the gait cycle events (heel strike and toe-off) were determined visually from the user during the post-processing analysis. This may cause some inconsistencies in the data, especially for the absolute angle values at foot strike, and possibly when reporting the range of motion for all of stance phase.

The Oxford foot model is not referenced to a measured neutral stance, but instead allows non-zero joint angle in the static reference position. This contributes to some of the increase in variability, as discussed in previous literature [16, 18]. However, this non-zero neutral position allows for a static foot posture measurement for persons with foot deformities where a neutral position in a static standing posture in a ‘zero’ position (i.e. foot flat on the ground) is not always possible, such as in the assessment of paediatric forefoot varus [5].

Future studies for repeatability of the OFM can include comparisons of genders, and participants with varying activity levels, recorded by the researchers. Increased amounts of between-day and within-session variability would be expected in pathological populations.

## Conclusions

The intra-rater reliability of the Oxford Foot Model was determined while walking in a neutral cushioning shoe in a healthy adult population. Between-day reliability ICCs were comparable to those reported previously for adults walking barefoot and shod. Bland-Altman plots for each joint showed good agreement overall for testing between days, and best with the relative measures such as excursion and range of motion. Within-session variability, evaluated with ICCs, were comparable to existing literature and demonstrated excellent reliability. This work shows that the OFM can produce reliable data when applied to the assessment of a shod foot.

## Abbreviations

ICC: Intraclass Correlation Coefficient
OFM: Oxford Foot Model
ROM: Range of Motion
SD: Standard Deviation
SEM: Standard Error of Measurement
